# Advancing cardiac xenotransplantation: overcoming challenges and ethical considerations for clinical integration

**DOI:** 10.3389/frtra.2026.1971770

**Published:** 2026-10-05

**Authors:** Ahmed A. Abdelrehim, Daniel J. Hurst, W. Hampton Gray, Robert Sorabella, Luz A. Padilla

**Affiliations:** 1 Department of Surgery, University of Alabama at Birmingham School of Medicine, Birmingham, AL, United States; 2 Department of Medical Education & Scholarship, Rowan-Virtua School of Osteopathic Medicine, Stratford, NJ, United States

**Keywords:** antibody-mediated rejection, cardiac xenotransplantation, clinical integration, congenital heart disease, health equity, research ethics

## Abstract

Advanced heart failure affects over 6 million adults in the United States alone. Orthotopic heart transplantation remains the definitive treatment, with over 90% one-year survival and meaningful gains in functional status and quality of life. The therapy is, however, constrained by an intractable supply problem. For decades, xenotransplantation (XTx), the transplantation of organs across species, has been proposed as a means of closing this gap, though for most of that time it remained a theoretical aspiration. With the discovery of CRISPR-Cas9 gene editing, improved immunosuppressive strategies, and refined preclinical models, the field has accelerated at an unprecedented pace. Multi-gene-edited porcine donors have survived for more than 200 days in orthotopic non-human primate models, and two compassionate-use pig-to-human cardiac transplants at the University of Maryland (2022 and 2023) demonstrated some feasibility for clinical cardiac XTx for the first time. In May 2026, the FDA granted clearance under an investigational new drug application to allow the EXPRESS study, the first prospective clinical trial of a gene-edited porcine heart, to proceed. In this perspective, we discuss the ethical, regulatory, and equity considerations that remain as decisive barriers to clinical integration and may shape how this therapy is introduced along the remaining immunologic and virologic challenges. We outline what we regard as the near-term priorities: defining the antibody targets that drive xenograft rejection, standardizing virologic surveillance, candidate selection and outcome benchmarking, and building access patterns with embedded distributive justice from the outset of its implementation.

## Introduction

Advanced heart failure affects over 6 million adults in the United States alone. Of these, an estimated 5% to 10% have advanced disease refractory to guideline-directed medical therapy (1). Yet fewer than 4,000 donor hearts become available for transplantation each year (2). Mechanical circulatory support devices extend survival for many of these patients, but device-related complications, anatomic contraindications, and limited applicability to certain populations leave a substantial unmet need. For decades, xenotransplantation (XTx), the transplantation of organs across species, has been proposed as a means of closing this gap but remained perpetually deferred in the field of cardiothoracic surgery. From early attempts in the 1960s and 1970s yielding only hours to weeks of graft survival, the field entered a prolonged period of dormancy after the Baby Fae baboon heart transplant in 1984 triggered public backlash (3, 4).

The subsequent shift to porcine donors and incremental advances in genetic engineering like CRISPR-Cas9 mediated knockout of immunogenic carbohydrate antigens and added human transgenes, and immunosuppression therapies eventually produced multi-gene-edited porcine donors capable of sustaining life-supporting cardiac function in non-human primates for months reinvigorating the XTx field (5, 6). These preclinical successes led to ground-breaking experiences in 2022 by the University of Maryland, implanting 10-gene-edited porcine hearts under compassionate use authorization, marking the first pig-to-human cardiac transplants in history (7, 8). Although neither recipient survived beyond two months, together with parallel advances in kidney XTx, these cases catalyzed the publication of the 2026 ISHLT Consensus Statement on Clinical Cardiac XTx, establishing criteria for recipient selection, immunologic assessment, and outcome benchmarking, and most recently, the FDA clearance of the investigational new drug (IND) application for the first-ever cardiac XTx clinical trial, the EXPRESS study, in May 2026 (7–10).

This perspective discusses the ethical, regulatory, and equity considerations that remain as barriers to clinical integration and may shape how this therapy is introduced along the remaining immunologic and virologic challenges. We attempt to synthesize the challenges to clinical integration, highlighting that these challenges are both biological and ethical, which should not be addressed separately but rather as a single, coupled agenda.

## Biological barriers to xenotransplantation

The immune response to a porcine xenograft is distinct from the alloimmune response that governs rejection of human donor organs. Allotransplant rejection is driven primarily by T-cell recognition of mismatched human leukocyte antigens, xenotransplant rejection involves multiple overlapping humoral, complement-mediated, cellular, and innate response that must be addressed simultaneously (11).

### Hyperacute rejection and xenoantigens

Mediated by preformed natural antibodies targeting carbohydrate antigens on the porcine vascular endothelium, this is considered to be the most immediate threat to a xenotransplanted heart. Three antigens have been identified as the principal targets: Galactose-alpha-1,3-galactose (alpha-Gal), the dominant antigen, produced by the enzyme alpha-1,3-galactosyltransferase encoded by the GGTA1 gene; N-glycolylneuraminic acid (Neu5Gc) antigen produce by CMAH gene; and the Sda antigen, encoded by B4GALNT2. Humans lack alpha-Gal and Neu5Gc and may carry preformed antibodies against these and Sda antigens. Anti-alpha-Gal antibodies can bind porcine endothelium and trigger complement-dependent cytotoxicity within minutes of reperfusion (12–15). CRISPR-Cas9 mediated knockout of all three genes, the triple knockout (TKO), has therefore become a foundation of modern porcine donor genetic engineering.

### Complement activation and coagulation dysregulation

Presents an independent barrier to xenograft survival. Complement regulatory proteins are species-specific: porcine complement regulators such as CD46, CD55, and CD59 do not effectively inhibit human complement components, triggering amplification of the classical and alternative complement pathways on the xenograft endothelium, producing membrane attack complex formation, endothelial activation, and microvascular thrombosis (16). Molecular incompatibilities between porcine and human coagulation factors, for example, porcine thrombomodulin can be a poor activator of human protein C, and porcine tissue factor pathway inhibitor does not adequately suppress human factor Xa, creating a coagulation dysregulation at the xenograft endothelial surface (17). Insertion of human transgenes encoding thrombomodulin (TBM), endothelial protein C receptor (EPCR), and tissue factor pathway inhibitor (TFPI) has been pursued to restore coagulation homeostasis across the species barrier.

### Antibody-mediated rejection

When hyperacute rejection is prevented through genetic modification and perioperative complement inhibition, the xenograft can remain vulnerable to antibody-mediated rejection (AMR), a process that unfolds over days to weeks and may have contributed to graft failure in both human cardiac xenotransplant expanded access recipients (7, 8). AMR in the xenotransplant setting involves both preformed and elicited antibodies, and hence recipients are selected with low baseline anti-pig antibody titers. They also differ in timeline and histologic pattern from the AMR seen in allotransplantation. AMR titers are followed closely as progressive antibody-mediated injury can lead to xenograft failure and may require different strategies for antibody-depleting therapies (18).

### T-cell mediated rejection and innate immune responses

While less dominant than AMR, remains a clinically relevant threat. The adaptive cellular immune response to xenografts involves both direct and indirect pathways of T-cell activation. Of particular interest, the species-specific interaction between porcine CD47 and human signal regulatory protein alpha (SIRP*α*). CD47, which serves as an inhibitory signal to macrophages; however, porcine CD47 is less efficient at engaging human SIRP*α*, rendering xenograft cells vulnerable to phagocytic clearance (19). Insertion of a human CD47 transgene into porcine donors is now standard in most multi-gene edited pig lines to address this incompatibility.

## First clinical experiences in cardiac xenotransplantation

The porcine donors used in the first human cardiac xenotransplants featured 10 genetic modifications designed to address the major immunologic and physiologic barriers to XTx as described above (7, 20). Four genes were inactivated: GGTA1, CMAH, and B4GALNT2, the triple knockout that eliminates the three principal carbohydrate xenoantigens, and the growth hormone receptor (GHrKO), intended to limit post-transplant xenograft overgrowth. Six human transgenes were inserted: two complement regulatory proteins (CD46 and CD55/DAF) to inhibit complement-mediated injury; two anticoagulant genes (thrombomodulin and endothelial protein C receptor) to mitigate coagulation dysregulation at the graft endothelial surface; CD47, to provide a signal that prevents macrophage-mediated phagocytosis; and heme oxygenase-1 (HO-1), an anti-inflammatory and anti-apoptotic protein intended to reduce ischemia-reperfusion injury and early graft inflammation. This 10-gene edited platform represented the most extensively modified porcine donor tested in any transplant setting and set the standard for subsequent clinical and preclinical XTx programs (Figure 1).

**Figure 1:**
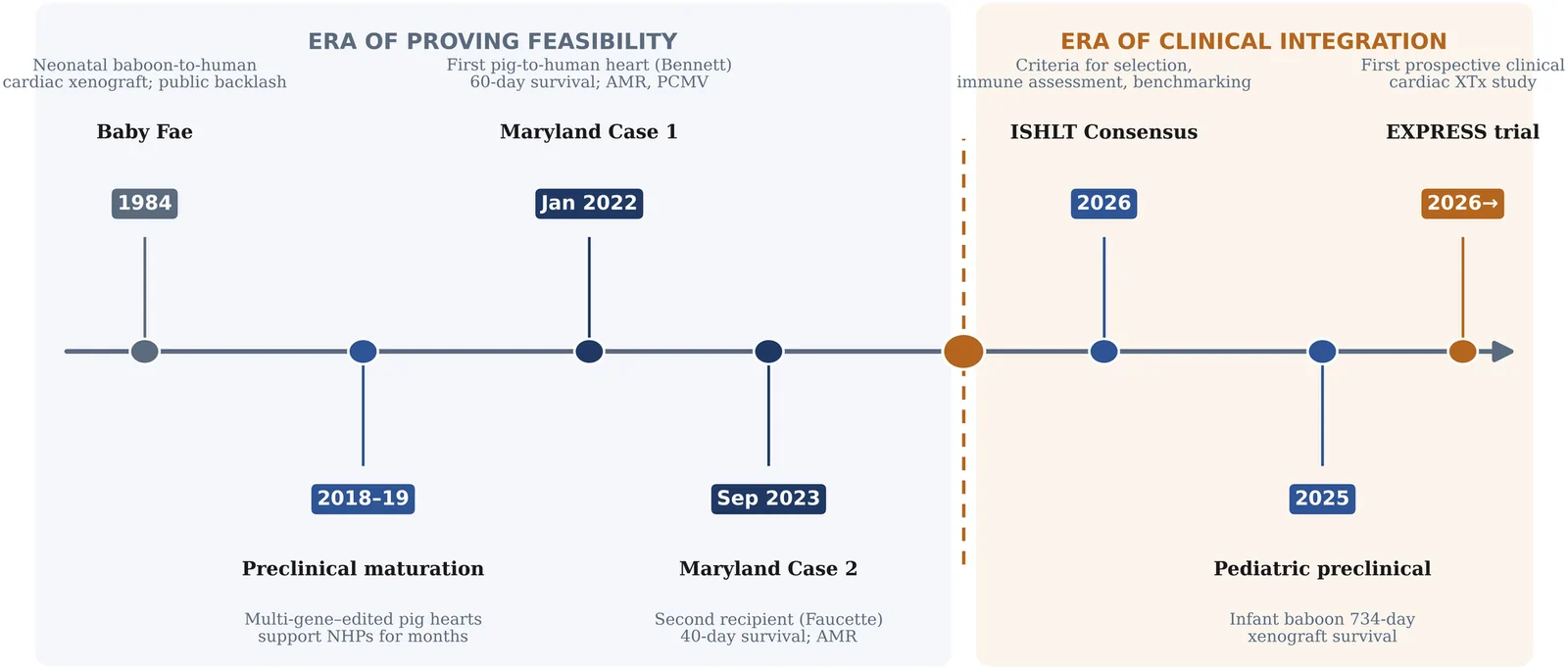
Timeline showing important milestones in cardiac xenotransplantation in the United States.

### Recipient 1 (January 7th, 2022)

On January 7, 2022, the University of Maryland performed the first pig-to-human orthotopic cardiac transplant under FDA expanded-access authorization (7). The first recipient was 57 year old, had terminal heart failure, was ineligible for allotransplantation or durable mechanical circulatory support, and had been on venoarterial ECMO for 48 days. The xenograft was preserved by non-ischemic continuous perfusion (NICP) with XVIVO perfusate, and immunosuppression comprised anti-CD40 costimulation blockade, rabbit antithymocyte globulin, anti-CD20, corticosteroids, and mycophenolate mofetil. The graft recovered, and the patient was weaned off ECMO by postoperative day 4 with a 55% ejection fraction, remaining hemodynamically stable with excellent graft function and no inotropic requirement for 47 days. On day 20, porcine cytomegalovirus (pCMV) was detected by cell-free DNA despite negative pre-transplant donor screening; the patient became neutropenic with severe hypogammaglobulinemia, prompting mycophenolate discontinuation and intravenous immunoglobulin (IVIG). He developed diastolic dysfunction requiring reinstitution of ECMO on day 49, and support was withdrawn on day 60 (7, 21). Retrospective analysis showed widespread endothelial injury consistent with AMR; several IVIG preparations bound donor aortic endothelial cells, raising the possibility that IVIG itself contributed to endothelial injury. The combination of pCMV reactivation, known to reduce xenograft survival in non-human primates, and possible IVIG-related injury may have contributed to the late graft deterioration (21, 22).

### Recipient 2 (September 20th, 2023)

The second recipient was 58-year-old, and similar to recipient 1 approved under FDA expanded access but was a healthier, limitedly ambulatory patient with deteriorating heart failure who was likewise not a candidate for allotransplantation or mechanical circulatory support (8). With enhanced pCMV surveillance combining quantitative PCR and serologic assays, no pCMV was detected at any point post-transplant (23). The xenograft was preserved with 49 min of NICP, and initial systolic and diastolic function were normal. On day 13, biopsy showed small areas of endothelial activation and prominent capillary antibody and complement staining (IgG, IgM, C3d, C4d) without endothelial injury. Despite low donor-specific anti-pig antibodies, the team initiated therapeutic plasma exchange and gave fresh frozen plasma, units that later tested positive for anti-pig antibodies. Acute hemodynamic decompensation occurred on day 29, requiring ECMO by day 31. The day 30 biopsy showed increasing capillary antibody and complement levels, diffuse endothelial activation, apoptotic endothelial cells, and myocyte ischemia; transcriptomic phenotyping alluded to AMR. Support was withdrawn on day 40. At autopsy, the xenograft had nearly doubled in weight (273 to 480 grams), with widespread endothelial injury and myocyte necrosis (8).

## What the first human experiences settled and what they did not

The authors believe that these experiences answered a foundational question that haunted the field since the 1960s, whether a genetically modified pig heart can physiologically sustain a human, with an affirmative answer.

First, the 10-gene-edited porcine heart is capable of immediate life-supporting function in human recipients and without hyperacute rejection. Second, both cases identified antibody-associated endothelial injury as a major threat to durable xenograft function, but they do not establish a single common mechanism of graft failure. In the first recipient, findings were compatible with AMR, but graft dysfunction was likely multifactorial, with potential contributions from pCMV reactivation, alterations in immunosuppression, and exposure to anti-pig antibodies contained in IVIG. In the second recipient, serial histopathologic, immunologic, and molecular findings suggested a more progressive AMR as a mechanism of graft failure. Residual antibody reactivity to porcine antigens not eliminated by current donor gene editing may be considered as a mechanism for xenograft injury, as relevant antigenic specificities and their causal contribution to graft injury are yet to be fully explored. Defining clinically relevant antibody targets could inform future donor engineering, crossmatch strategies, antibody surveillance, and interpretation of xenograft pathology (7, 8, 11). Third, pCMV surveillance protocols should be rigorously standardized, as latent virus undetectable by conventional screening may reactivate with devastating consequences. Fourth, the administration of blood products including IVIG and fresh frozen plasma to xenotransplant recipients should consider careful pre-screening for anti-pig antibody. Lastly, xenograft overgrowth remains a concern despite growth hormone receptor knockout, suggesting that additional strategies, such as the use of inherently smaller donor breeds like Yucatan miniature pigs may be explored as an approach to control graft size.

None of these pivotal unresolved facts of the field is a reason for pessimism; but each is a well defined target. However, each also means that a recipient enrolled in EXPRESS today is being asked to accept a therapy whose dominant failure mode is understood in outline but not in mechanism, a fact with direct ethical weight, to which we describe below.

## The scientific priority: from feasibility to durability

The two cases established clear feasibility but left a new question of how to maintain the graft. Hence, our focus next turns to durability, which, in the authors’ opinion, focuses on three key priorities: 1) The antibody targets that drive xenograft AMR must be defined at the epitope level. The non-Gal, non-Neu5Gc antigens responsible for graft loss remain unidentified. As a result, the next generation of gene edits cannot be directed at the specific targets driving rejection, and crossmatch and antibody-monitoring assays cannot be built to detect the relevant antibodies or to distinguish which titers actually threaten the graft. Characterizing these targets is, in our view, one of the highest yield investments the field can make; as it simultaneously informs the next generation of gene edits, the appropriate genetically-engineered pig model, the design of crossmatch assays, and the interpretation of every trial biopsy (11); 2) Immunosuppression must be optimized for the xenogeneic context rather than carried over unchanged from allotransplantation. As outlined, the AMR observed in the Maryland cases differed in timeline and histologic pattern from the AMR seen in allotransplantation, raising questions about whether standard AMR therapies will be sufficient in the xenotransplant setting. Current strategies under investigation include enhanced B-cell depletion with anti-CD20 monoclonal antibodies (rituximab), downstream complement inhibition (eculizumab), and costimulation blockade with anti-CD154 or anti-CD40 agents to prevent T-cell dependent antibody maturation, which has been central to the longest preclinical survivals. Investigational agents such as tegoprubart, an anti-CD40L antibody, offer potential additional advantage of avoiding calcineurin-inhibitor nephrotoxicity (6); 3) Virologic surveillance must be standardized. The contrast between the two cases supports rigorous donor screening and post-transplant virologic surveillance. PCMV reactivation may have complicated the first recipient, whereas no evidence of pCMV replication was detected in the second recipient after more sensitive donor screening. Although this observation cannot establish that enhanced screening prevented reactivation, it supports the value of multimodal screening strategies capable of detecting latent infection that may be missed by conventional PCR alone (8, 22). In adult animals, viral load often falls below the PCR detection limit during latency, necessitating complementary serological assays for reliable detection. While pCMV risk can be reduced through rigorous multimodal donor screening, porcine endogenous retrovirus (PERV) risk can be more difficult to mitigate prospectively. PERV is different from an exogenous latent pathogen such as pCMV because PERV sequences are integrated into the porcine genome. However, PERV can be characterized through donor selection and virologic testing, but uncertainty regarding transmission and long-term consequences can persist. Which shifts the surveillance question from the individual to the public and carries scientific and ethical public health implications, with the possibility of genomic integration and potential consequences such as altered gene regulation or oncogenesis. The role of PERV in humans remains uncertain despite the absence of documented transmissions. Ongoing surveillance using quantitative nucleic acid testing, whole genome sequencing, and metagenomic approaches will be essential as clinical xenotransplantation programs expand.

## Preclinical progress in pediatric models

A distinctive and, we would argue, underappreciated line of progress comes from the pediatric preclinical work. At the 2026 ISHLT meeting, a Children’s Hospital Los Angeles group reported an infant baboon that survived more than two years (734 days) with a functioning heart from a genetically edited Yucatan miniature pig, far exceeding any prior orthotopic cardiac xenograft survival (10, 24). The result matters beyond the pediatric domain for two reasons. It supports that graft overgrowth can be controllable when the donor breed is chosen for a slower growth trajectory, and it shows that durability is achievable. The neonatal immune window, relative lack of preformed anti-pig antibodies, and the amenability of the infant immune system to tolerization, first suggested by Baby Fae, may make congenital heart disease one of the most scientifically favorable settings for XTx. However, it is also among the most ethically demanding, involving a vulnerable population with one of the greatest unmet needs for donor organs (25).

## The ethical priority is not downstream of science

A common implicit model holds that science must mature first and the ethics will follow to regulate a proven therapy. However, the ethical challenges facing cardiac XTx are not ancillary to its success; they may determine it as the field moves into prospective trials (26).

Consider informed consent. EXPRESS will enroll patients aged 50 or older with stage D heart failure and no remaining therapeutic options, by design, individuals facing near-certain mortality (9). Cardiac XTx uniquely tests the limits of acceptable uncertainty in surgical innovation as a research team cannot fully disclose a risk profile it does not yet understand. Furthermore, candidates are typically critically ill, often dependent on temporary mechanical support, and face imminent mortality. This clinical context introduces a well-described vulnerability to therapeutic misconception, the tendency to conflate experimental intervention with individualized clinical care (27). Ethical guidance increasingly emphasizes that consent processes must go beyond disclosure to actively counteract this misconception. This includes explicit articulation that the primary purpose of early trials is knowledge generation rather than therapeutic benefit, as well as transparency regarding the limited survival observed to date. Independent consent monitors and staged consent processes have been proposed to mitigate coercion, particularly when patients are approached under expanded access pathways (28).

The FDA IND clearance for the EXPRESS trial marks a transition from compassionate use to systematic investigation. However, the boundary between these pathways remains ethically fraught. Expanded access may generate limited publicly available scientific knowledge and providing insufficient data to assess efficacy or safety. Conversely, adverse outcomes in individual expanded-access cases may receive substantial attention and potentially jeopardize progression too, or public confidence in formal clinical trials. This tension underscores the importance of balancing timely access for individual patients with transparency, systematic data collection, and preservation of scientific rigor. Consensus recommendations for trial design emphasize several key principles: restriction to highly selected candidates, standardized immunologic and infectious risk assessment, and incorporation of adaptive trial structures that allow protocol modification in response to early outcomes. Independent oversight, through data and safety monitoring boards and external ethics committees, is essential to maintain public trust and ensure that enthusiasm does not outpace evidence (28).

A related challenge is recipient selection. Criteria based on “last-resort” status are ethically defensible but may inadvertently concentrate risk in the sickest patients, potentially obscuring signals of efficacy. Balancing fairness, scientific validity, and patient welfare remains an unresolved tension in early-phase XTx trials (29).

Zoonotic risk extends the ethical circle beyond the recipient entirely, as explained above. Recipients may be asked to accept lifelong surveillance, mandatory reporting, biobanking, and constraints on certain activities as conditions of transplantation (30). These obligations complicate the conventional right to withdraw from research, particularly because infectious-disease surveillance may remain necessary even after xenograft failure or removal. Protocols and consent processes should therefore distinguish between research procedures from which participants may withdraw and surveillance obligations considered necessary to protect public health, while defining how conflicts or nonadherence will be managed (27). These obligations may also extend to close contacts, who may require education and, in the event of suspected xenozoonotic transmission, testing, monitoring, or quarantine. Long-term surveillance and biobanking further create substantial confidentiality and data-governance obligations, including prospective specification of specimen retention, future use, data access, and circumstances in which public-health responsibilities may limit conventional expectations of privacy (28). These competing obligations cannot be resolved on a case by case at the bedside; they require standing transparent governance, international coordination, and regulatory standards established before broad deployment.

Pediatric XTx amplifies these concerns because potentially lifelong obligations may be initiated before recipients can make an autonomous decision. Enrollment requires parental permission together with age and developmentally appropriate assent when the child is capable of providing it. As pediatric recipients mature, ongoing assent and eventual re-consent should be incorporated into longitudinal follow-up, particularly because surveillance obligations may extend into adulthood. A maturing recipient may ultimately object not only to continued surveillance but also to living with a porcine xenograft that was accepted on their behalf. In that setting, refusal of immunosuppression or other required care could become a means of expressing that objection and may intentionally or unintentionally precipitate graft rejection. This possibility is particularly relevant given the recognized challenges of treatment and follow-up adherence during adolescence after transplantation. These scenarios create a distinctive tension between developing autonomy, preservation of graft function, and the continuing public-health obligations associated with XTx when applied to a vulnerable population.

Finally, a parallel obligation runs to the source animals themselves; the routine use of extensively gene-edited pigs raises welfare and animal stewardship concerns that differ from conventional agricultural use in scale and intent. Maintaining public trust will require rigorous and transparent standards governing donor animal breeding, housing, genetic modification, and the minimization of suffering, while also addressing broader concerns regarding environmental sustainability and responsible resource utilization (31).

## Equity must be designed in, not retrofitted

Of the ethical dimensions, distributive justice is the one most consequential to defer. Early XTx is resource-intensive, it requires designated-pathogen-free donor animals, specialized facilities, complex immunosuppression, and concentrated institutional expertise. Left to develop without deliberate policy, these features will tend to localize access within a small number of elite centers, and among patients who can navigate them could exacerbate existing disparities in advanced heart failure care (26, 31). Stakeholder analyses highlight the importance of integrating equity considerations early in development, including transparent allocation frameworks, payer engagement, and strategies to avoid concentration of access within a small number of elite centers (26, 31). It would be difficult to retrofit once access patterns have set.

## Discussion

Our position is that the ethical challenges of clinical integration are coupled and must be pursued in parallel (Table 1). With the FDA IND clearance for the first cardiac XTx clinical trial, the field continues to face not only the remaining immunologic and virologic hurdles but also the ethical, regulatory, and equity considerations that will shape how this therapy is introduced, with neither domain advancing in isolation. Scientifically, the next phase will require iterative refinement of donor genotypes to eliminate residual xenoantibody targets, optimization of costimulation blockade based immunosuppression, and rigorous standardization of virologic surveillance, particularly for latent porcine pathogens. However, technical progress alone will be insufficient to ensure adoption. Equally critical is the development of a durable ethical and regulatory infrastructure that can support first-in-human and early-phase trials without compromising scientific validity or public trust. This includes stringent patient selection frameworks grounded in high unmet need, standardized and transparent consent processes that mitigate therapeutic misconception, and internationally harmonized oversight of zoonotic risk. Adaptive trial designs and disciplined use of expanded access pathways will be essential to balance innovation with evidence generation. Finally, proactive attention to distributive justice must shape early implementation. Without deliberate policy, XTx risks reinforcing existing disparities in advanced heart failure care into a scalable, equitable, and societally adopted therapeutic paradigm.

**Table 1:** The coupled scientific and ethical challenges of clinical integration. Each unresolved biological problem generates a corresponding ethical obligation; progress on one reduces the burden of the other.

Challenge	Scientific dimension	Coupled ethical/integration dimension
Antibody-mediated rejection	Non-Gal, non-Neu5Gc target epitopes remain uncharacterized; potential contributory cause of graft loss in both human cases	Failure mode cannot be fully disclosed in consent or interpreted on biopsy until targets are defined
Immunosuppression	Optimal regimen for human recipients is undefined; allograft-derived AMR therapy may not translate to xenograft	Novel-agent toxicity and uncertainty during informed consent
Virologic risk	PCMV risk can be mitigated through rigorous/standardized donor screening; PERV can be characterized through donor/product testing, but transmission and long-term consequences uncertainty remains.	Lifelong surveillance, mandatory reporting, and activity constraints extend obligation to public health
Xenograft overgrowth	Persists despite GHR knockout; consideration of smaller donor breeds to mitigate	Bears directly on suitability for growing pediatric recipients who cannot consent
Candidate selection	Last-resort cohorts concentrate competing mortality, obscuring efficacy signals	Risks concentrating experimental risk in vulnerable groups
Access	Resource-intensive donor supply, facilities, and expertise	Distributive justice must be designed early to prevent exacerbating existing disparities

PCMV, porcine cytomegalovirus; PERV, porcine endogenous retrovirus; GHR, growth hormone receptor; AMR, antibody-mediated rejection.

## Data Availability

The original contributions presented in the study are included in the article/supplementary material, further inquiries can be directed to the corresponding author.

## References

[1] Metra M Ponikowski P Dickstein K McMurray JJV Gavazzi A Bergh C-H. Advanced chronic heart failure: a position statement from the study group on advanced heart failure of the heart failure association of the European Society of Cardiology. Eur J Heart Fail. (2007) 9:684–94. 10.1016/j.ejheart.2007.04.003 17481947

[2] Colvin MM Smith JM Ahn YS Messick E Lindblad K Israni AK. OPTN/SRTR 2021 annual data report: heart. Am J Transplant. (2023) 23(2 Suppl 1):S300–78. 10.1016/j.ajt.2023.02.008 37132346

[3] Hardy JD Chavez CM Kurrus FD Neely WA Eraslan S Turner MD. Heart transplantation in man. Developmental studies and report of a case. JAMA. (1964) 188:1132–40. 10.1001/jama.1964.03060390034008 14163110

[4] Bailey LL Nehlsen-Cannarella SL Concepcion W Jolley WB. Baboon-to-human cardiac xenotransplantation in a neonate. JAMA. (1985) 254:3321–9. 10.1001/jama.1985.03360230053022 2933538

[5] Längin M Mayr T Reichart B Michel S Buchholz S Guethoff S. Consistent success in life-supporting porcine cardiac xenotransplantation. Nature. (2019) 568:E7. 10.1038/s41586-019-1108-4 30944481

[6] Mohiuddin MM Singh AK Corcoran PC Thomas III ML Clark T Lewis BG. Chimeric 2C10R4 anti-CD40 antibody therapy is critical for long-term survival of GTKO.hCD46.hTBM pig-to-primate cardiac xenograft. Nat Commun. (2016) 7:11138. 10.1038/ncomms11138 27045379 PMC4822024

[7] Griffith BP Goerlich CE Singh AK Rothblatt M Lau CL Shah A. Genetically modified porcine-to-human cardiac xenotransplantation. N Engl J Med. (2022) 387:35–44. 10.1056/NEJMoa2201422 35731912 PMC10361070

[8] Griffith BP Grazioli A Singh AK Tully A Galindo J Saharia KK. Transplantation of a genetically modified porcine heart into a live human. Nat Med. (2025) 31:589–98. 10.1038/s41591-024-03429-1 39779924

[9] United Therapeutics Corporation. United Therapeutics Corporation announces FDA clearance to proceed with UHeart xenotransplantation clinical trial (EXPRESS). Press Release (2026). Available online at: https://ir.unither.com/press-releases/2026/05-15-2026-230017490 (Accessed April 29, 2026).

[10] Mehra MR Mohiuddin MM Reichart B Abicht J-M Boulet J Cooper DKC. The 2026 international society for heart and lung transplantation consensus statement on clinical cardiac xenotransplantation. J Heart Lung Transplant. (2026) 45(4):e129–47. 10.1016/j.healun.2025.12.012 41718516

[11] Sykes M Sachs DH. Transplanting organs from pigs to humans. Sci Immunol. (2019) 4:eaau6298. 10.1126/sciimmunol.aau6298 31676497 PMC7293579

[12] Galili U. Anti-gal: an abundant human natural antibody of multiple pathogeneses and clinical benefits. Immunology. (2013) 140:1–11. 10.1111/imm.12110 23578170 PMC3809700

[13] Sandrin MS McKenzie IF. Gal alpha(1,3)Gal, the major xenoantigen(s) recognised in pigs by human natural antibodies. Immunol Rev. (1994) 141:169–90. 10.1111/j.1600-065x.1994.tb00877.x 7532618

[14] Varki A. Colloquium paper: uniquely human evolution of sialic acid genetics and biology. Proc Natl Acad Sci U S A. (2010) 107(Suppl 2):8939–46. 10.1073/pnas.0914634107 20445087 PMC3024026

[15] Byrne GW Du Z Stalboerger P Kogelberg H McGregor CG. Cloning and expression of porcine beta1,4 N-acetylgalactosaminyl transferase encoding a new xenoreactive antigen. Xenotransplantation. (2014) 21:543–54. 10.1111/xen.12124 25176027 PMC4262693

[16] Dalmasso AP Vercellotti GM Fischel RJ Bolman RM Bach FH Platt JL. Mechanism of complement activation in the hyperacute rejection of porcine organs transplanted into primate recipients. Am J Pathol. (1992) 140(5):1157–66. PMID: 1580328 1580328 PMC1886521

[17] Cowan PJ Robson SC d'Apice AJF. Controlling coagulation dysregulation in xenotransplantation. Curr Opin Organ Transplant. (2011) 16:214–21. 10.1097/MOT.0b013e3283446c65 21415824 PMC3094512

[18] Singh AK Goerlich CE Shah AM Zhang T Tatarov I Ayares D. Cardiac xenotransplantation: progress in preclinical models and prospects for clinical translation. Transpl Int. (2022) 35:10171. 10.3389/ti.2022.10171 35401039 PMC8985160

[19] Ide K Wang H Tahara H Liu J Wang X Asahara T. Role for CD47-SIRPalpha signaling in xenograft rejection by macrophages. Proc Natl Acad Sci U S A. (2007) 104:5062–6. 10.1073/pnas.0609661104 17360380 PMC1829264

[20] Pierson RN Fishman JA Lewis GD D’Alessandro DA Connolly MR Burdorf L. Progress toward cardiac xenotransplantation. Circulation. (2020) 142:1389–98. 10.1161/CIRCULATIONAHA.120.048186 33017208 PMC7990044

[21] Mohiuddin MM Singh AK Scobie L Goerlich CE Grazioli A Saharia K. Graft dysfunction in compassionate use of genetically engineered pig-to-human cardiac xenotransplantation: a case report. Lancet. (2023) 402:397–410. 10.1016/S0140-6736(23)00775-4 37393920 PMC10552929

[22] Denner J Längin M Reichart B Krüger L Fiebig U Mokelke M. Impact of porcine cytomegalovirus on long-term orthotopic cardiac xenotransplant survival. Sci Rep. (2020) 10:17531. 10.1038/s41598-020-73150-9 33067513 PMC7568528

[23] Halecker S Hansen S Krabben L Ebner F Kaufer B Denner J. How, where and when to screen for porcine cytomegalovirus (PCMV) in donor pigs for xenotransplantation. Sci Rep. (2022) 12:21545. 10.1038/s41598-022-25624-1 36513687 PMC9747970

[24] Cleveland JD Mitchell CB Swicord W Neal SJ Vo C Bakshi K. Gene-edited pig cardiac xenotransplantation as a bridge to allotransplantation in infants: progress in a pig-to-baboon model. Am J Transplant. (2026) 26(5):980–91. 10.1016/j.ajt.2025.12.017 41453738 PMC12935157

[25] Cleveland DC Banks CA Hara H Carlo WF Mauchley DC Cooper DKC. The case for cardiac xenotransplantation in neonates: is now the time to reconsider xenotransplantation for hypoplastic left heart syndrome? Pediatr Cardiol. (2019) 40:437–44. 10.1007/s00246-018-1998-1 30302505

[26] Khush KK Bernat JL Pierson RN Silverman HJ Parent B Glazier AK. Research opportunities and ethical considerations for heart and lung xenotransplantation research: a report from the national heart, lung, and blood institute workshop. Am J Transplant. (2024) 24:918–27. 10.1016/j.ajt.2024.03.015 38514013 PMC11144553

[27] Hurst DJ Padilla LA Walters W Hunter J Cooper DKC Eckhoff DM. Paediatric xenotransplantation clinical trials and the right to withdraw. J Med Ethics. (2019) 46:311–5. 10.1136/medethics-2019-105668 31704780

[28] Padilla LA Hurst D Maxwell K Gawlowicz K Paris W Cleveland D. Informed consent for potential recipients of pig kidney xenotransplantation in the United States. Transplantation. (2022) 106:1754–62. 10.1097/TP.0000000000004144 35475475 PMC10124773

[29] Hurst DJ Padilla L. Ethically advancing pediatric cardiac xenotransplant. JAMA Pediatr. (2024) 178:5–6. 10.1001/jamapediatrics.2023.4681 37955921

[30] Hurst DJ Padilla LA Rodger D Schiff T Cooper DKC. Close contacts of xenograft recipients: ethical considerations due to risk of xenozoonosis. Xenotransplantation. (2024) 31(2):e12847. 10.1111/xen.12847 38468191

[31] Hurst DJ Bobier C Padilla LA. Ethical issues involved in solid organ xenotransplantation. Clin Anat. (2026) 39:55–9. 10.1002/ca.70036 40965314 PMC12747646

